# Extremely Low-Frequency Electromagnetic Fields Affect Transcript Levels of Neuronal Differentiation-Related Genes in Embryonic Neural Stem Cells

**DOI:** 10.1371/journal.pone.0090041

**Published:** 2014-03-03

**Authors:** Qinlong Ma, Ping Deng, Gang Zhu, Chuan Liu, Lei Zhang, Zhou Zhou, Xue Luo, Min Li, Min Zhong, Zhengping Yu, Chunhai Chen, Yanwen Zhang

**Affiliations:** Department of Occupational Health, Faculty of Preventive Medicine, Third Military Medical University, Chongqing, People's Republic of China; Hospital Nacional de Parapléjicos - SESCAM, Spain

## Abstract

Previous studies have reported that extremely low-frequency electromagnetic fields (ELF-EMF) can affect the processes of brain development, but the underlying mechanism is largely unknown. The proliferation and differentiation of embryonic neural stem cells (eNSCs) is essential for brain development during the gestation period. To date, there is no report about the effects of ELF-EMF on eNSCs. In this paper, we studied the effects of ELF-EMF on the proliferation and differentiation of eNSCs. Primary cultured eNSCs were treated with 50 Hz ELF-EMF; various magnetic intensities and exposure times were applied. Our data showed that there was no significant change in cell proliferation, which was evaluated by cell viability (CCK-8 assay), DNA synthesis (Edu incorporation), average diameter of neurospheres, cell cycle distribution (flow cytometry) and transcript levels of cell cycle related genes (P53, P21 and GADD45 detected by real-time PCR). When eNSCs were induced to differentiation, real-time PCR results showed a down-regulation of Sox2 and up-regulation of Math1, Math3, Ngn1 and Tuj1 mRNA levels after 50 Hz ELF-EMF exposure (2 mT for 3 days), but the percentages of neurons (Tuj1 positive cells) and astrocytes (GFAP positive cells) were not altered when detected by immunofluorescence assay. Although cell proliferation and the percentages of neurons and astrocytes differentiated from eNSCs were not affected by 50 Hz ELF-EMF, the expression of genes regulating neuronal differentiation was altered. In conclusion, our results support that 50 Hz ELF-EMF induce molecular changes during eNSCs differentiation, which might be compensated by post-transcriptional mechanisms to support cellular homeostasis.

## Introduction

Exposure to extremely low frequency electromagnetic fields (ELF-EMFs) from power lines and consumer devices has progressively increased over the past 30-years [1]. This has provoked widespread public concern about the possible effects of ELF-EMF on human health. Epidemiologic surveys have pointed the possible association of the augmented risk of brain tumors [2], and Alzheimer's disease [3], with exposure to ELF-EMF from overhead power lines and various consumer devices. Therefore, it is necessary to investigate and understand the potential effects on central nervous system.

A great deal of evidence has confirmed that ELF-EMF can affect the central nervous system. *In vivo*, ELF-EMF exposure improved social recognition memory [4] and promoted recovery of sensorimotor behavior in rats with hemisection of thoracic spinal cord [5]. However, another study revealed an increased level of apoptotic cells in mouse brains [6]. *In vitro*, exposure of PC12 cells to ELF-EMF significantly suppressed or promoted neuronal differentiation in different culture conditions, whereas cell division was not changed [7]. Lisi A et al. reported that exposure to 50 Hz ELF-EMF promoted early maturation and differentiation in newborn rat cerebellar granule neurons [8]. The stress proteins, cytoskeletal protein levels and proliferation of cultured astroglial cells were not altered after exposure to ELF-EMF (50 Hz, 1 mT) [9]. Because of contradictory results, the potential adverse effects of ELF-EMF exposure on the CNS remain an area of intense research.

As we know, during pregnancy, the developing brain is acutely sensitive to exogenous physical and chemical agents. Embryonic neural stem cells (eNSCs), which can generate neurons, astrocytes and oligodendrocyte cells, provide a unique model to evaluate the influence of genetic or environmental factors on embryonic development *in vitro*. Their proliferation and differentiation will affect the number of NSCs and neurons in each region of the brain [10]. A recent study showed a significantly enhanced proliferation in human neuroblastoma after exposure to a continuous magnetic field (50 Hz, 1 mT) [11]. Moreover, continuous exposure to ELF-EMFs (50 Hz, 1 mT) promoted neuronal differentiation of postnatal NSCs *in vitro* [12] and adult neurogenesis *in vivo* [13]. However, there have been few studies about the effects of 50 Hz ELF-EMF exposure on the embryonic NSCs.

The aim of the present study was to determine whether intermittent exposure to 50 Hz ELF-EMF affected proliferation and differentiation of eNSCs. We found that 50 Hz ELF-EMF exposure with different magnetic intensities and different exposure time did not affect cell proliferation. Although the percentages of neurons (Tuj1 positive cells) and astrocytes (GFAP positive cells) differentiated from eNSCs were not changed, the transcript levels of some early genes related to neuronal differentiation were significantly altered, indicating that intermittent exposure to 50 Hz ELF-EMF had the ability to induce changes at the transcript level during eNSCs differentiation, yet showed no central hazardous effect on the proliferation and neuronal differentiation of eNSCs.

## Materials and Methods

### Ethics Statement

This study was approved by the Animal Research Committee of the Third Military Medical University. The animal care and procedures were in accordance with its guidelines.

### Embryonic NSCs culture and differentiation

Telencephalons were dissected from embryonic day 13.5 (E13.5) BALB/c mice, and eNSCs were prepared as previously described [14]. The cells were cultured in a complete medium containing a 1∶1 (v/v) mixture of Dulbecco's modified Eagle's medium (DMEM) and F12 medium (Gibco, USA), epidermal growth factor (EGF) (20 ng/mL; Sigma-Aldrich, USA), basic fibroblast growth factor (bFGF) (20 ng/mL; Sigma-Aldrich, USA), and B27 (1×) supplements (Gibco, USA), held at 37°C in a humidified atmosphere in the presence of 5% CO_2_. At 3-day intervals, half of the volume of media was replaced. To induce differentiation, eNSCs were plated onto coverslips or Flasks pre-coated with Poly-L-lysine (Sigma, USA) in a differentiation medium, in which bFGF and EGF had been replaced with 1% fetal bovine serum (FBS) (HyClone, USA).

### ELF-EMF exposure

The exposure system was built and provided by the Foundation for Information Technologies in Society (IT'IS foundation, Zurich, Switzerland). Detailed information of this system was described in Ref [15]. Briefly, the setup generated a vertical EMF, composed of two four-coil systems (two coils with 56 windings, two coils with 50 windings), and was placed inside a μ-metal chamber. Both chambers were placed inside a commercial incubator (HERAcell® 150 i, Thermo Scientific, USA) to ensure stable environmental conditions (37°C, 5% CO_2_, 95% humidity). The exposure set-up was controlled and monitored by a computer through specific sensors. During exposure, the temperature in the chambers was monitored and maintained at 37.0±0.5°C. The temperature variance between the chambers did not exceed 0.3°C.

All the experiments were performed at a frequency of 50 Hz sinusoidal waves with magnetic intensities of 0.5 mT, 1 mT and 2 mT for 3 days; or with a magnetic intensity of 2 mT for 1 day, 2 days and 3 days, with an intermittent cycle of 5 min on/10 min off under double blind conditions, while sham and exposure groups were randomly selected by the computer.

### Cell viability and DNA synthesis analysis

The cell viability was assessed using a colorimetric cell counting kit (CCK-8; Dojindo, Japan). According to the manufacturer's instructions, eNSCs were cultured in a 96-well plate (1.0×10^5^ cells/ml) and then exposed to 50 Hz ELF-EMF at various magnetic intensities of 0.5 mT, 1 mT and 2 mT for 3 days; or were exposed to 50 Hz ELF-EMF at a magnetic intensity of 2 mT for 1 day, 2 days and 3 days. Then, 10 µl of CCK-8 solution was added into the 100 µl of medium for 3 h at 37°C. The absorbance was measured at 450 nm with a microplate reader (SPECTRAFLUOR, TECAN, Sunrise, Austria). In each experiment, six paralleled wells were made. The DNA synthesis of eNSCs was studied using a Cell-Light EdU DNA cell proliferation kit according to the manufacturer's instructions (Guangzhou RiboBio, Guangzhou, China). Briefly, eNSCs incubated in 35 mm Petri-dishes in proliferation medium were exposed to 50 Hz ELF-EMF at a magnetic intensity of 2 mT for 1 day, 2 days and 3 days, respectively. In the last 24 h, cells were exposed to EdU (20 µM). Cells were dissociated and plated onto pre-coated coverslips with poly-L-lysine at a density of 1.0×10^5^ cells/ml after the EMF exposure. eNSCs were fixed for 20 min with 4% paraformaldehyde after attachment for 6 h. Then, cells were rinsed twice with PBS and stained by incubating for 30 min with the staining mix. After staining, the cells were washed 3 times with TBS with 0.5% Triton X-100. Cell nuclei were counterstained with Hoechst 33342 (5 µg/mL; Sigma-Aldrich, USA). The number of EdU-positive and total cells was counted using fluorescence microscopy (Leica, Germany) in four non-overlapping fields per coverslip.

### Diameter Measuring of Neurospheres

eNSCs were incubated in 35 mm Petri-dishes in proliferation medium and exposed to 50 Hz ELF-EMF at a magnetic intensity of 2 mT for 3 days. The pictures of neurospheres were obtained using an inverted microscope (Leica, Germany) at the end of exposure in at least 5 fields per dish. The diameters of neurospheres were measured through the Image J software.

### Cell cycle analysis

For cell cycle analysis, the eNSCs were harvested after 50 Hz ELF-EMF treatment (2 mT, 5 min on/10 min off for 3 days), digested into single cells with accutase (eBioscience, USA), washed three times with cold PBS and fixed in 75% ice-cold ethanol overnight at 4°C. Then, the fixed cells were stained with 50 µg/ml propidium iodide (PI) containing 50 µg/ml RNase A (DNase free) for 30 min at room temperature with protection from light and analyzed in a flow cytometer.

### Immunofluorescence assays

Neurospheres that were cultured for 6 days *in vitro* were separated into single cells using accutase (eBioscience, USA), and plated onto poly-L-lysine-coated glass coverslips at a density of 1.0×10^4^/cm^2^ for 24 h in proliferation medium. After adhesion, the medium was replaced with differentiation medium and the cells were treated with ELF-EMF exposure. For immunocytochemistry, cells were fixed with 4% paraformaldehyde for 20 min at room temperature (RT), washed twice in PBS, and permeabilized with 0.3% TritonX-100 for 10 min. Cells were then blocked with 0.3% bovine serum albumin (BSA) for 30 min and incubated overnight (at 4°C) with primary antibodies against Nestin(mouse,1∶100, Chemicon, USA), Tuj1 (mouse, 1∶100, R&D, USA) and GFAP (rabbit, 1∶200, Beijing Zhongshan, Beijing, China). The following day, cells were washed twice with PBS and incubated for 1 h at RT with secondary antibodies: donkey anti-mouse Alexa Fluor® 488 (1∶100, Invitrogen, USA) and chicken anti-rabbit Alexa Fluor® 647 (1∶100, Invitrogen, USA). Hoechst 33342 (5 µg/mL; Sigma-Aldrich, USA) was used to counterstain the cell nucleus. The Tuj1 positive cells and GFAP positive cells were counted in four different fields of each coverslip for each experiment using fluorescence microscopy (Leica, Germany) at magnification of 630, and data were expressed as percentages of the total number of cells within the same field.

### Reverse-transcription PCR and real-time PCR analysis

The expression of genes was detected according with Liu Yao et al [16]. After 50 Hz ELF-EMF exposure, total RNA was extracted using the Trizol reagent (Takara, Japan). The cDNAs were obtained by reverse transcription-PCR (RT-PCR) kit (TOYOBO, Japan). Then the expression of interest genes was examined through a Bio-Rad IQ5 Detection System with the SYBR Green PCR Master mix (TOYOBO, Japan). The GAPDH was used to be an internal control in quantitative analysis. Gene-specific primers were used to amplify P53 (5′-GAG GCC GGC TCT GAG TAT ACC A-3′ and 5′-GGC AGG CAC AAA CAC GAA CC-3′), P21 (5′-CCA ATC CTG GTG ATG TCC GA-3′ and 5′-AGT CAA AGT TCC ACC GTT CTC G-3′), GADD45 (5′-GCT GGC TGC TGA CGA AGA C-3′ and 5′-CGG ATG AGG GTG AAA TGG AT-3′), Sox2 (5′-AAC CGA TGC ACC GCT ACG A-3′ and 5′-TGC TGC GAG TAG GAC ATG CTG-3′), Tuj1 (5′-CGC CAT GTT CAG ACG CAA G-3′ and 5′-CTC GGA CAC CAG GTC GTT CA-3′) and GFAP (5′-AGC CAA GGA GCC CAC CAA AC-3′ and 5′-TCT ATA CGC AGC CAG GTT GTT CTC-3′), Ngn1 (5′-ATC ACC ACT CTC TGA CCC-3′), Ngn2 (5′-GTC ATC CTC CAA CTC CAC GTC-3′ and 5′-AGG CGC ATA ACG ATG CTT CTC-3′), Math1 (5′-GAG TGG GCT GAG GTA AAA GAG T-3′ and 5′-GGT CGG TGC TAT CCA GGA G-3′), Math3 (5′-CTC TTA TGG AAT GCT CGG AAC C-3′ and 5′ AAT CTT TCA AGG CGA GCT TTA GTC-3′), Hes1 (5′-GAA GAG GCG AAG GGC AAG AA-3′ and 5′- GAG GTG CTT CAC AGT CAT TTC CA-3′), Hes5 (5′-GAC CGC ATC AAC AGC AGC AT-3′ and 5′-GGC GAA GGC TTT GCT GTG T-3′), Hes6 (5′- CCT GGT GGA GAA GAA GCG AC-3′ and 5′-TTG GCC TGC ACC TCG GTA-3′), NeuroD (5′- ACA ACA GGA AGT GGA AAC ATG ACC-3′ and 5′-CAC TCA TCT GTC CAG CTT GGG-3′) and GAPDH (5′- ATA CGG CTA CAG CAA CAG GG-3′ and 5′- GCC TCT CTT GCT CAG TGT CC-3′) (Takara, Japan). The threshold cycle number (Ct) values of genes were determined. Gene expression level was normalized to GAPDH and presented as the fold change (2^−ΔΔCt^) above sham group: ΔΔCt = (Ct _selected gene_−Ct _GAPDH_)_exposed group_−(Ct _selected gene_−Ct _GAPDH_)_sham group_ [17].

### Statistics

All data were expressed as mean ± standard deviation (SD) from at least three independent experiments performed by duplication, unless otherwise stated. The Levene's test results indicated that the data showed homogeneity of variance. The differences between sham group and ELF-EMF group were made by the Student's *t*-test. Significant differences were established at *P*<0.05.

## Results

### Identification of the eNSCs

**Figure 1:**
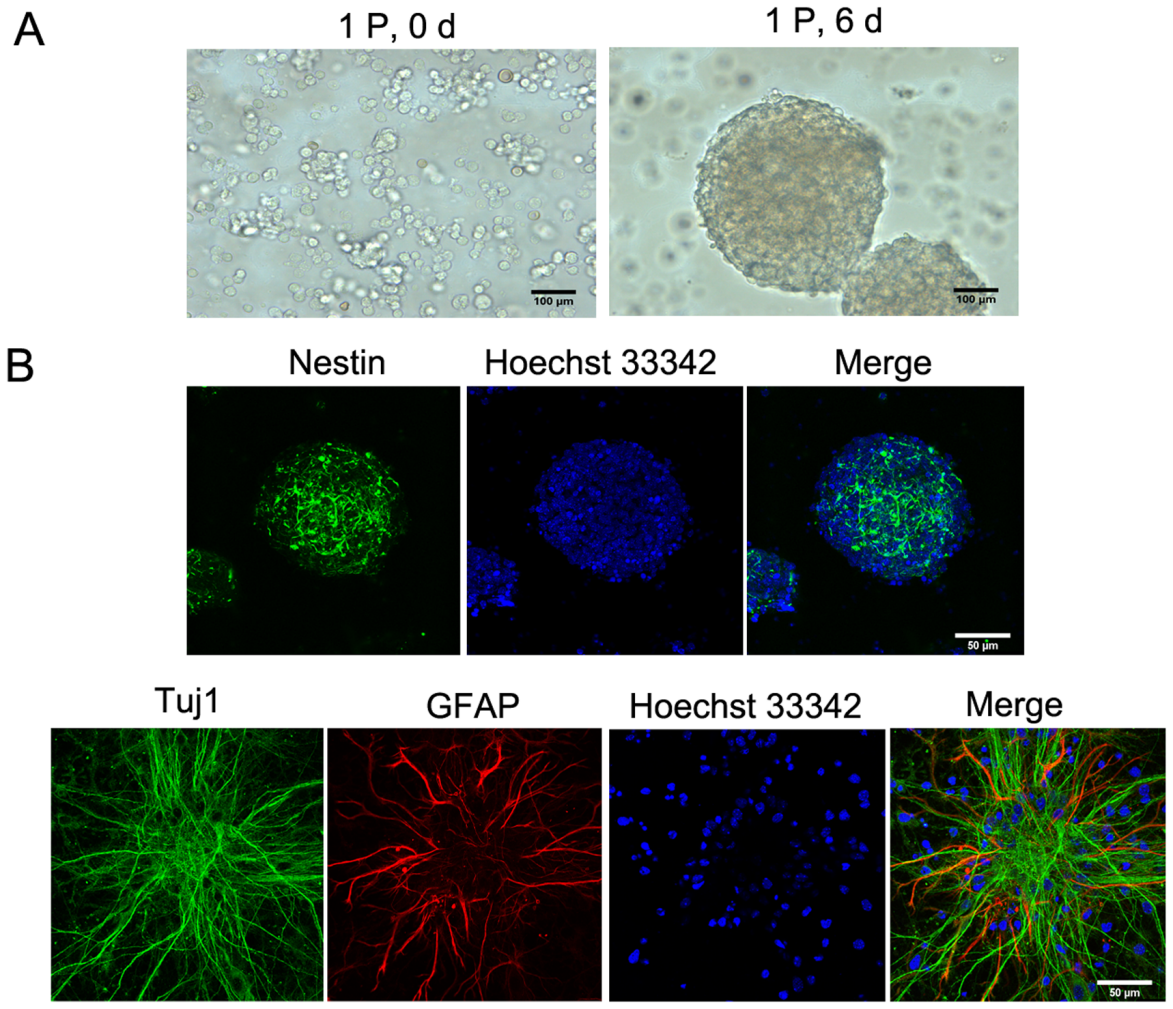
Identification of embryonic neural stem cells (eNSCs). A: The isolated single eNSCs formed neurospheres in proliferation medium. Left: new isolated single eNSCs (1P, 0 d) and right: neurospheres cultured for 6 days *in vitro* (1P, 6 d). P: passage. Scale bar, 100 µm. B: Neurospheres expressed nestin protein (NSCs specific protein) in proliferation medium and became positive for Tuj1 (a neuronal marker) and GFAP (a glial marker) by immunocytochemical assay cultured for 6 days in differentiation medium. Scale bar, 50 µm.

Firstly, the identification of the eNSCs was carried out. The new isolated single cells (1P, 0 d) from telencephalon were cultured in proliferation medium. The cells which had differentiated into neurons and glia cells died, whereas eNSCs proliferated and formed neurospheres (1P, 6 d) as shown in Fig. 1A, and were positive for nestin (NSCs specific protein) (Fig. 1B). For cell differentiation, neurospheres were cultured in differentiation medium. Neurospheres were positive for Tuj1 (a neuronal marker) and GFAP (an astrocyte marker) by immunocytochemistry as shown in Fig. 1B. Therefore, the neurosphere-like eNSCs preserved the ability of proliferation and differentiation.

### Effects of 50 Hz ELF-EMF exposure on the cell viability of eNSCs

In order to examine the effects of 50 Hz ELF-EMF exposure on the cell viability of eNSCs, eNSCs were exposed to 50 Hz ELF-EMF at different intensities of 0.5 mT, 1 mT and 2 mT for 3 days; or were exposed at an intensity of 2 mT for 1 day, 2 days and 3 days, with an intermittent cycle of 5 min on/10 min off. After exposure, the cell viability in different groups was detected by CCK-8 kit. Dose-dependent results showed no significant differences between sham- and exposed-groups (Table 1), and there was also no marked difference from time-dependent results (Table 2).

**Table 1:** The dose-dependent results of the cell viability of eNSCs between sham group and exposed group.

Groups Exp.	0.5 mT		1 mT		2 mT	
	Sham	Exposed	Sham	Exposed	Sham	Exposed
Exp.1	0.36±0.02	0.33±0.02	0.36±0.01	0.36±0.01	0.36±0.02	0.36±0.01
Exp.2	0.34±0.02	0.34±0.01	0.34±0.01	0.33±0.02	0.35±0.01	0.35±0.01
Exp.3	0.37±0.04	0.36±0.03	0.35±0.01	0.36±0.04	0.34±0.04	0.35±0.02
*P* a	0.83		0.29		0.47	
*P* b	0.26		0.72		0.95	

eNSCs were exposed to 50 Hz ELF-EMF at intensities of 0.5 mT, 1 mT and 2 mT for 3 days. The cell viability was examined by CCK-8 assay. Results were obtained from three independent experiments (Exp.1, Exp.2 and Exp.3) and the data were expressed as mean ± SD.

a The test for homogeneity of variance between sham groups and ELF-EMF groups was made by the Levene's test.

b The statistical evaluation between sham groups and ELF-EMF groups was made by the Student's *t*-test.

**Table 2:** The time-dependent results of the cell viability of eNSCs between sham group and exposed group.

Groups Exp.	1 day		2 days		3 days	
	Sham	Exposed	Sham	Exposed	Sham	Exposed
Exp.1	0.12±0.03	0.11±0.02	0.24±0.04	0.24±0.02	0.33±0.03	0.34±0.02
Exp.2	0.10±0.01	0.12±0.02	0.25±0.04	0.27±0.02	0.35±0.02	0.35±0.01
Exp.3	0.10±0.03	0.09±0.01	0.26±0.05	0.27±0.01	0.34±0.01	0.33±0.02
*P* a	0.65		0.23		1.00	
*P* b	1.00		0.44		1.00	

eNSCs were exposed to 50 Hz ELF-EMF at a magnetic intensity of 2 mT for 1 day, 2 days and 3 days. The cell viability was examined by CCK-8 assay. Results were obtained from three independent experiments (Exp.1, Exp.2 and Exp.3) and the data were expressed as mean ± SD.

a The test for homogeneity of variance between sham groups and ELF-EMF groups was made by the Levene's test.

b The statistical evaluation between sham groups and ELF-EMF groups was made by the Student's *t*-test.

### Effects of 50 Hz ELF-EMF exposure on the DNA synthesis of eNSCs

**Figure 2:**
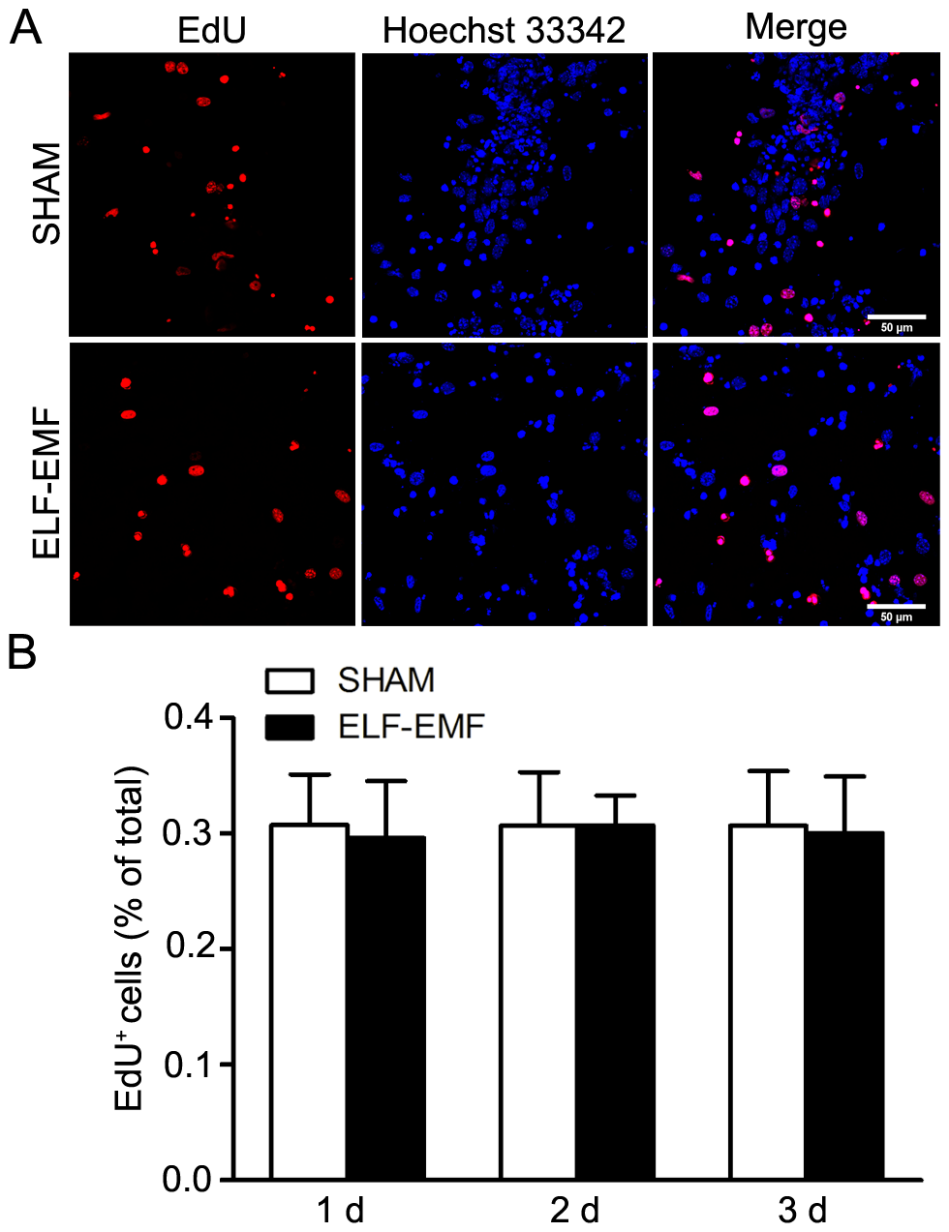
The effects of 50 Hz ELF-EMF on DNA synthesis of eNSCs detected by EdU incorporation. Neurospheres cultured in proliferation medium were exposed to 50 Hz ELF-EMF at a magnetic intensity of 2 mT for 1 day, 2 days and 3 days. EdU was added to the medium during the last 24 h. A: Representative images of double stained EdU/Ho 33342 cells. The percentage of EdU positive cells was quantified in four random fields in each culture well. Scale bar: 50 µm. B: The graph showed the statistical results. Results were obtained from three independent experiments and the data were expressed as mean ± SD.

Since no marked change of eNSCs viability was observed after 50 Hz ELF-EMF exposure at all-time point and applied magnetic field intensities, we proceeded to investigate the effects of ELF-EMF on DNA synthesis of eNSCs at the magnetic field intensity of 2 mT for 1 day, 2 days and 3 days, and the DNA synthesis was detected by EdU incorporation. As shown in Fig. 2, the percentage of EdU positive cells remained unchanged at all-time points, indicating 50 Hz ELF-EMF exposure did not affect DNA synthesis of eNSCs.

### Effects of 50 Hz ELF-EMF exposure on the average diameter of neurospheres

**Figure 3:**
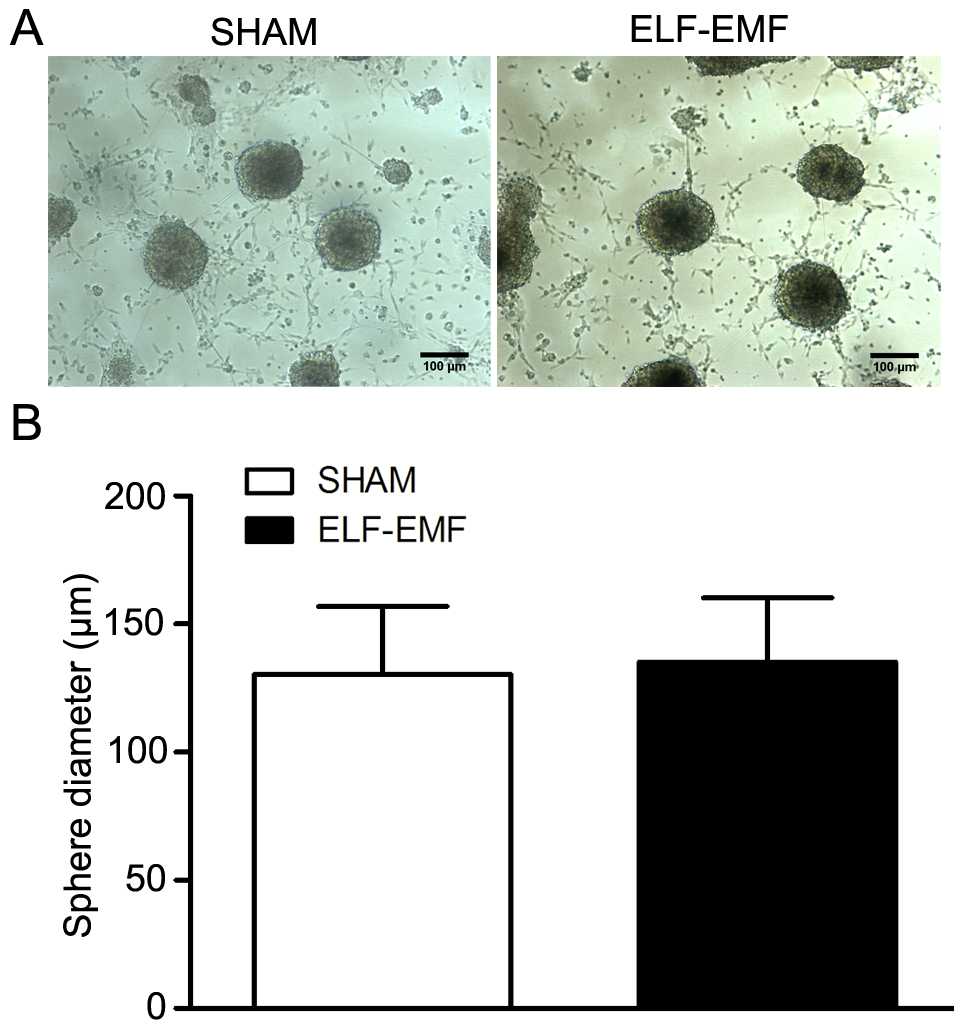
The effects of 50 Hz ELF-EMF on the average diameter of neurospheres. Neurospheres cultured for 6 days were dissected mechanically into small clusters, incubated in 35-dishes in proliferation medium and exposed to 50 Hz ELF-EMF (2 mT for 3 days). A: Representative images of neurospheres. Scale bar: 100 µm. B: The graph showed the statistical results. The diameters of neurospheres were measured by the Image J software. The data were expressed as mean ± SD from three independent experiments. The total number of measured neurospheres was 100 in sham groups and 145 in exposed groups.

Because the growth of eNSCs was also represented with the diameter of neurospheres, we measured the average diameter of neurospheres exposed to 50 Hz ELF-EMF. Primary eNSCs, cultured for 6 days, were dissected mechanically into small clusters and incubated in Petri-dishes in proliferation medium. The diameters of neurospheres were measured after 50 Hz ELF-EMF exposure (2 mT for 3 days). As shown in Fig. 3, we found no obvious distinction in the average diameter of neurospheres between sham- and exposed-groups.

### Effects of 50 Hz ELF-EMF exposure on the cell cycle and cell cycle-related genes of eNSCs

**Figure 4:**
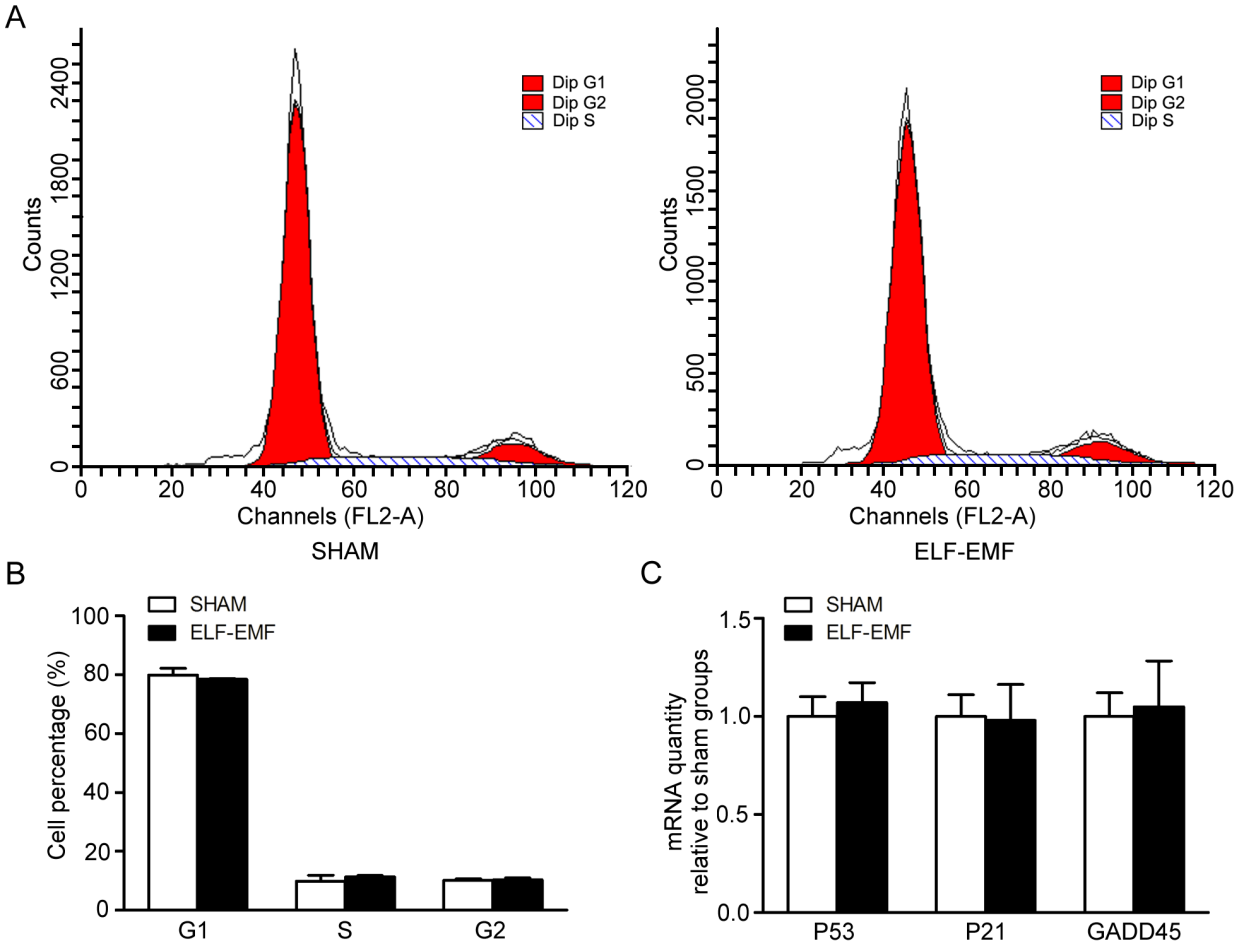
The effects of 50 Hz ELF-EMF exposure on the cell cycle and cell cycle-related genes of eNSCs. eNSCs were cultured in proliferation medium with 50 Hz ELF-EMF exposure (2 mT for 3 days). The fixed cells were stained with PI and analyzed in a flow cytometer. A and B: There was no significant change in cell cycle between sham- and exposed-groups. C: Expression of p53, p21, and GADD45 mRNA was detected by real-time PCR. The mouse GAPDH mRNA expression levels were used as endogenous reference. Gene expression level was normalized to GAPDH and presented as the fold change (2^−ΔΔCt^) above sham group. The data were expressed as mean ± SD from three independent experiments with three replicates per experiment.

The proliferation of eNSCs is also associated with the process of cell cycle. So the cell cycle was analyzed after 50 Hz ELF-EMF exposure (2 mT for 3 days) by a flow cytometer and no significant change was found in exposed-groups compared with sham-groups (Fig. 4A and B), and Fig. 4C showed that there was also no significant difference at the transcript level of cell cycle-related genes-p53, p21, and GADD45-after 50 Hz ELF-EMF exposure (2 mT for 3 days) by real-time PCR analysis.

### Effects of 50 Hz ELF-EMF exposure on the differentiation of eNSCs

**Figure 5:**
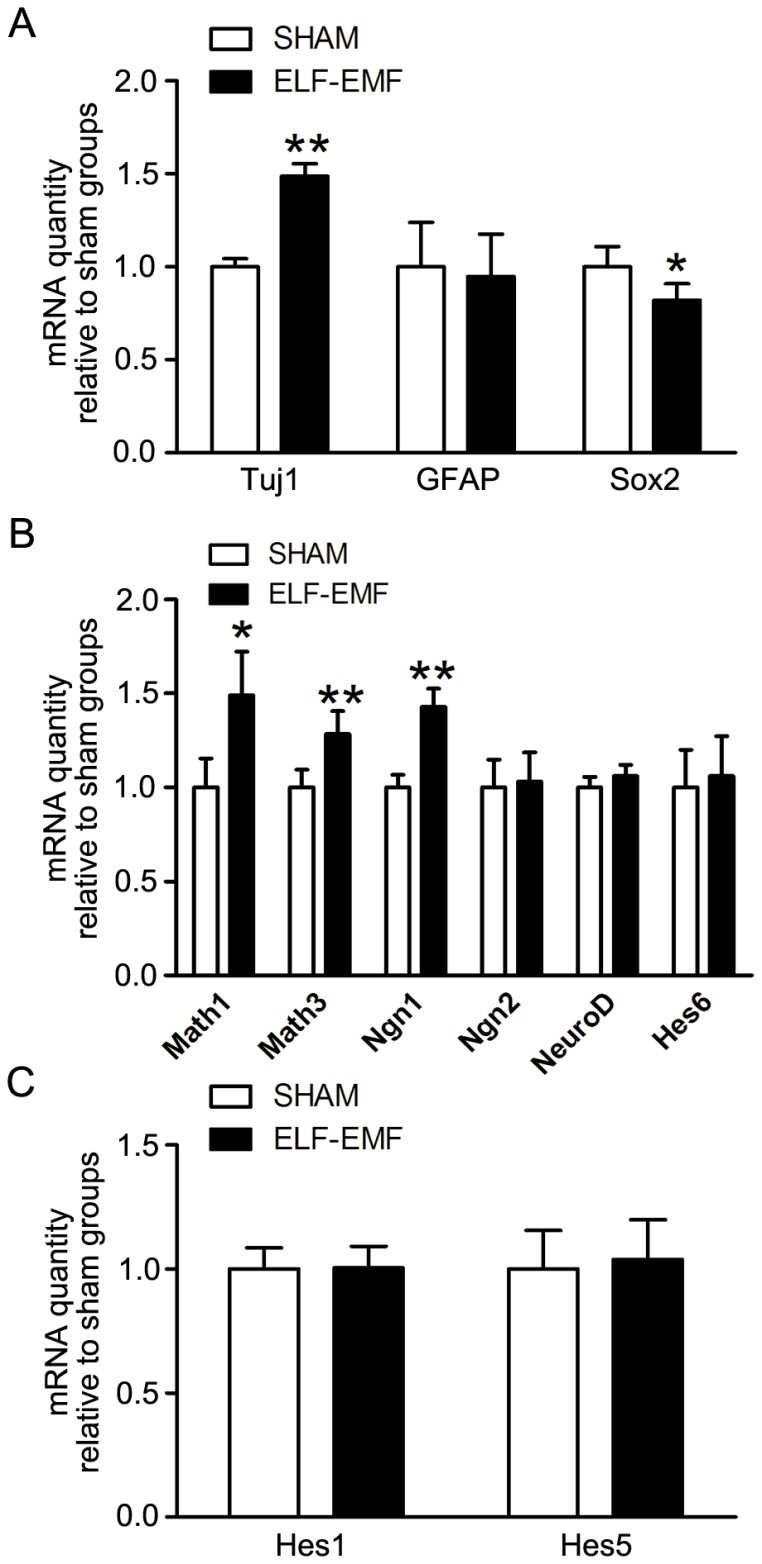
The effects of 50 Hz ELF-EMF exposure on the relative mRNA levels of genes regulating neuronal differentiation of eNSCs. eNSCs were cultured in differentiation medium with 50 Hz ELF-EMF exposure (2 mT for 3 days) and the expression of Tuj1, GFAP, and Sox2 in (A); Math1, Math3, Ngn1, Ngn2, NeuroD and Hes6 in (B); Hes1 and Hes5 in (C) mRNA was detected by real-time PCR. The mouse GAPDH mRNA expression levels were used as endogenous reference. Gene expression level was normalized to GAPDH and presented as the fold change (2^−ΔΔCt^) above sham group. The Ct values of GAPDH between sham groups and ELF-EMF groups were shown in Appendix S1. The data were expressed as mean ± SD from three independent experiments with three replicates per experiment. *P<0.05 and **P<0.01 as compared with sham groups.

To determine the effects of 50 Hz ELF-EMF exposure on cell differentiation, the relative mRNA levels of genes regulating cell differentiation were detected by real-time PCR analysis. Firstly, the eNSCs were harvested after 50 Hz ELF-EMF exposure (2 mT for 3 days) and real-time PCR analysis was carried out to detect the gene expression of Tuj1, GFAP and Sox2. As shown in Fig. 5A, an up-regulation of Tuj1 and down-regulation of Sox2 were observed after 50 Hz ELF-EMF exposure, but the mRNA expression of GFAP was not altered. Consequently, some early genes, which promote neuronal differentiation, were analyzed and an up-regulation of Math1, Math3 and Ngn1 was observed after 50 Hz ELF-EMF exposure (2 mT for 3 days), but the mRNA expression of Ngn2, Hes6 and NeuroD was not altered (Fig. 5B). At last, some early genes, which promote glia differentiation, were also analyzed. As shown in Fig. 5C, the mRNA expression of Hes1 and Hes5 did not change after 50 Hz ELF-EMF exposure (2 mT for 3 days). The Ct values of GAPDH between sham groups and ELF-EMF groups were shown in Appendix S1.

**Figure 6:**
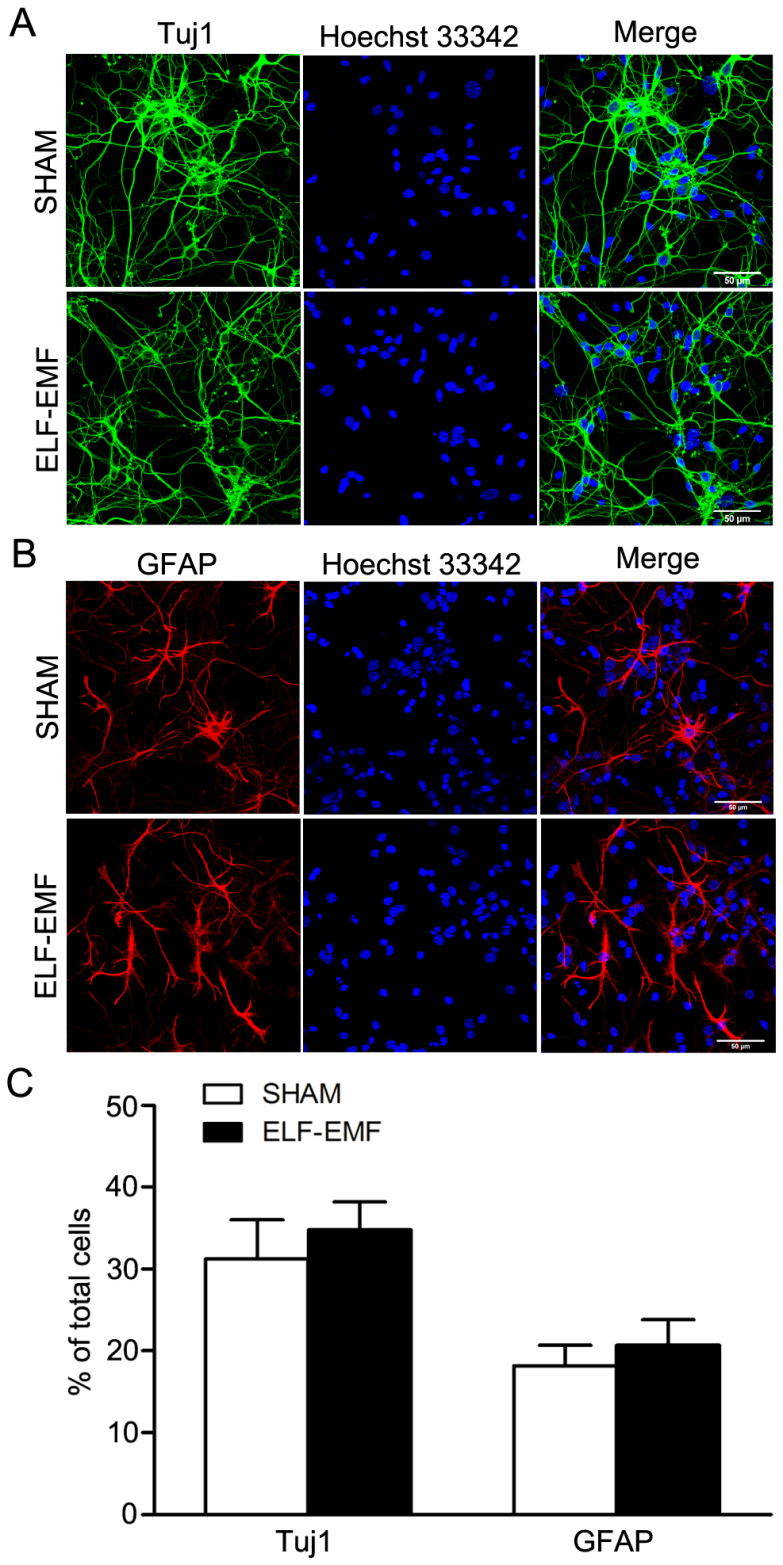
The effects of 50 Hz ELF-EMF exposure on the percentages of neurons and astrocytes differentiated from eNSCs. Neurospheres were dissociated into single cells and cultured in differentiation medium with 50 Hz ELF-EMF exposure (2 mT for 3 days). The differentiated cells were incubated with antibody against Tuj1 and GFAP. The images in (A) and (B) are representative of three independent experiments (Scale bar: 50 µm), and the graph in (C) shows the statistical results. The data were expressed as mean ± SD from three independent experiments.

Then the percentages of neurons (Tuj1 positive cells) and astrocytes (GFAP positive cells) differentiated from eNSCs were detected by immunofluorescence assay. After 50 Hz ELF-EMF exposure (2 mT for 3 days), both of the percentages of Tuj1 positive cells and GFAP positive cells showed no significant change as shown in Fig. 6.

These results suggest that 50 Hz ELF-EMF exposure may affect early gene expression of neuronal differentiation, although the percentage of neurons was not altered.

## Discussion

Here, our data showed that intermittent exposure to 50 Hz ELF-EMF did not alter cell viability, DNA synthesis, average diameter of neurospheres, cell cycle, mRNA expression of P53, P21 and GADD45 of eNSCs in proliferation medium. When eNSCs were induced to differentiation, 50 Hz ELF-EMF exposure resulted in a down-regulation of Sox2 and up-regulation of Math1, Math3, Ngn1 and Tuj1 mRNA levels, however, the percentages of Tuj1 positive cells and GFAP positive cells differentiated from eNSCs were not changed.

In present study, the magnetic intensity was chosen according to the guidelines recommended by International Commission on Non-Ionizing Radiation Protection (INIRC) in 1998 [18], in which the magnetic intensity of occupational exposure should not over 0.5 mT. In this study, the largest magnetic intensity was set up to 2 mT, four times of occupational exposure limits, which was also applied by Nikolova [19]. Moreover, the exposure time in most of studies was defined as population-doubling time of the selected cell type [20]. Therefore, in this study, the exposure time was set up to 3 days, about 2–3 times of the used cell's population-doubling time. Additionally, the adopted intermittent exposure with cycles of 5 min on and 10 min off in the present study was first studied by Ivancsits S [21], and then replicated in later studies [22], [23], which showed that it had a more powerful effect than continuous exposure to induce DNA strand breaks. It is well known that DNA damage may induce changes in cell cycle arrest, cell proliferation, apoptosis, differentiation and other cell biology [24]–[27], and few studies have been conducted to apply this intermittent exposure to eNSCs. Therefore, it is meaningful to unveil possible adverse reactions of 50 Hz ELF-EMF on eNSCs using this exposure mode.

In proliferative cells, CCK-8 assay was used to evaluate not only cell viability [28], but also indirectly to reflect cell numbers or proliferation [29]. Combination with DNA synthesis detected by EdU incorporation (a classic method for proliferation assay), and dose-dependent and time-dependent results, showed that the proliferation of eNSCs was not influenced after 50 Hz ELF-EMF exposure. Nikolova's study [19] revealed that intermittent exposure to ELF-EMF (50 Hz, 2 mT, 5 min on and 30 min off) did not affect mitochondrial function, cell cycle and cell proliferation in ES-derived neural progenitor cells, which was similar to our findings. However, other studies confirmed that continuous ELF-EMF (50 Hz, 1 mT) exposure enhanced proliferation of human neuroblastoma [11] and postnatal mouse NSCs *in vitro* [13]. Therefore, different magnetic intensities and exposure mode (continuous versus intermittent) may result in different results.

Previous studies provide evidence that the cell cycle control plays a capital role in regulating the proliferative expansion and self-renewal capacity of neural progenitor cells [30]–[32]. So the distribution of cell cycle is involved in proliferation of eNSCs. Moreover, P53 can regulate G1/S arrest through its downstream-P21 and GADD45, following gamma-irradiation of human lymphoma cells [33]. In our experiments, real-time PCR analysis revealed no obvious change at transcript level of P53, P21 and GADD45 in proliferative eNSCs, and consistent results in percentage of different cell cycle phases detected by a flow cytometer. Jaroslaw Czyz [34] reported that 50 Hz ELF-EMF exposure to p53-deficient embryonic stem cells (ES) with magnetic intensity of 2.3 mT at an intermittent scheme of 5 min on/30 min off induced a significant up-regulation of transcript levels of the egr-1, paralleled by a transient up-regulation of mRNA levels of p21 and c-jun, but not in wide type ES cells. However, ELF-EMF exposure with intermittent cycles of 5 min on/10 min off had no effect on gene expression levels of egr-1, p21 and c-jun in undifferentiated wide type or p53^−/−^ ES cells, which conformed to our results. Meanwhile, with respect to morphology, neurosphere size also reflects the proliferative ability of NSCs [35]. No significant alteration of the diameter of neurospheres was observed between sham- and exposed-groups in our study. These findings indicate that there were no effects of 50 Hz ELF-EMF intermittent exposure on eNSCs proliferation under our conditions.

It is well known that the balance between proliferation and differentiation in NSCs is critically controlled by diverse factors. Sox2 is implicated in controlling neural progenitor maintenance [36], [37], and progenitor commitment requires Sox2 repression to allow transcription of proneural factors [38], [39]. Temporo-spatial regulation of neuronal differentiation is critically controlled by diverse bHLH genes in neural stem cells. These include the repressor type (Hes1 and Hes5) and the activator type (Math1, Math3, Ngn1, Ngn2, Hes6 and NeuroD) [40], [41]. We observed a down-regulation of Sox2, which suggests a transcription of proneural factors that is consistent with an up-regulation of activator type bHLH (Math1, Math3, Ngn1) and Tuj1 at the transcript level. However, the ratio of neurons (Tuj1 positive cells) was not changed by immunocytochemistry. Additionally, the selected repressor type bHLH genes (Hes1 and Hes5) were also not altered, which is consistent with the mRNA expression of GFAP and the ratio of astrocytes (GFAP positive cells). Therefore, we speculated that intermittent exposure to 50 Hz ELF-EMF would partially affect neuronal differentiation at the transcript level. Although other studies showed that continuous exposure to ELF-EMF (50 Hz, 1 mT) promoted neuronal differentiation of NSCs *in vitro* [12] and neurogenesis *in vivo* [13], we observed no alteration on the percentage of Tuj1 positive cells. Similarly to our data, there was no alteration of the transcript levels of central nervous system specific genes in newborn rats after maternal continuous exposure to 60 Hz magnetic fields *in vivo* [42]. Nikolova [17] also reported that 50 Hz ELF-EMF exposure to ES-derived neural progenitor cells with magnetic intensity of 2 mT at an intermittent scheme of 5 min on/30 min off induced no changes of neuron (Nurr1, TH)- and glial (GFAP)-specific genes at the transcript and protein level.

In present study, we found an up-regulation of Math1, Math3, Ngn1 and Tuj1 at mRNA level, but the percentages of Tuj1 positive cells and GFAP positive cells did not change, suggesting some compensatory mechanisms at the translational and posttranslational level may account for this. Additionally, Tuj1, a cytoskeleton protein in neurons, is also used to evaluate axon growth and maturation of neurons [43]. This indicates that intermittent exposure to 50 Hz ELF-EMF may be associated with axon growth and maturation of neurons under our conditions, which needs further research.

Last but not least, we should realize that the inconsistent biological effects of EMF exposure may come from the differences in exposure sources, experimental protocols, and biological systems. Moreover, this weak effect may be observed through more susceptible experimental methods and the keen observation of laboratory assistants.

In short, we conclude that intermittent exposure of 50 Hz ELF-EMF affected the mRNA levels of genes related to neuronal differentiation of eNSCs *in vitro*. However, since there were no detectable changes in cell proliferation, or in the percentage of neuronal differentiation, we speculate that ELF-EMF responses at the transcript level may be compensated at the posttranscriptional level and (or) associated with maturation of neurons. Such claims need further investigation.

## Supporting Information

Appendix S1 **The Ct values of GAPDH between sham groups and ELF-EMF groups in** **Figure 5** **.**
(DOC)
