# Supplementary material for: Extremely Low-Frequency Electromagnetic Fields Affect Transcript Levels of Neuronal Differentiation-Related Genes in Embryonic Neural Stem Cells
Source: PLoS One. 2014 Mar 3;9(3):e90041. doi: 10.1371/journal.pone.0090041 (PMC3940726; doi:10.1371/journal.pone.0090041)
Supplement: Appendix S1 — The Ct values of GAPDH between sham groups and ELF-EMF groups in Figure 5 . (DOC) [file pone.0090041.s001.doc]

**Table S1.** The Ct values of GAPDH between sham groups and ELF-EMF groups in Figure 5.

| Groups  Exp. | Sham | | | Exposed | | |
| --- | --- | --- | --- | --- | --- | --- |
| Exp.1 | 15.29 | 15.31 | 15.27 | 15.38 | 15.41 | 15.39 |
| Exp.2 | 14.57 | 14.59 | 14.60 | 14.53 | 14.52 | 14.49 |
| Exp.3 | 16.13 | 16.16 | 16.10 | 16.22 | 16.19 | 16.20 |

Differentiating eNSCs were exposed to 50 Hz ELF-EMF at an intensity of 2 mT for 3 days. The Ct values of GAPDH between sham groups and ELF-EMF groups were obtained from three independent experiments (Exp.1, Exp.2 and Exp.3) with three replicates per experiment.
